# Magnet Aspiration Into the Distal Lung of a Pediatric Patient: A Case Report

**DOI:** 10.7759/cureus.66567

**Published:** 2024-08-10

**Authors:** Meera R Laxman, Santino S Cervantes, Christie G Cherian, Lena Naffaa, Tamarah Westmoreland

**Affiliations:** 1 Pediatric Surgery, Nemours Children's Health, Orlando, USA; 2 Pediatric Otolaryngology, Nemours Children's Health, Orlando, USA; 3 Pediatric Pulmonology, Nemours Children's Health, Orlando, USA; 4 Pediatric Radiology, Nemours Children's Health, Orlando, USA; 5 Pediatric Surgery, University of Central Florida (UCF) College of Medicine, Orlando, USA

**Keywords:** pediatric surgery, multidisciplinary management, pediatric lung, ball magnet, magnet aspiration

## Abstract

Foreign body (FB) aspiration is one of the most common life-threatening emergencies in children and one of the leading causes of mortality in the pediatric population. Most commonly, aspirated items are organic materials, such as nuts and seeds. Inorganic objects are usually plastic or metal. Symptoms of aspiration can vary depending on the location, area and amount of blockage, and object size and shape. Because of the difficult airway anatomy of children, a multidisciplinary approach - including otolaryngology, pulmonology, anesthesia, and general surgery - for the removal of airway FBs is necessary and prudent to avoid more invasive surgical involvement. This report discusses a nine-year-old male who aspirated two ball magnets, which became lodged in his tracheobronchial tree and required a multidisciplinary approach for removal.

## Introduction

One of the most common life-threatening emergencies in children is foreign bodies (FBs) stuck in the airway, either organic or inorganic [1-4]. Within the pediatric population, tracheobronchial FBs are among the leading causes of mortality [4,5]. Due to their narrower and shorter upper airway, underdeveloped protective airway mechanisms, and lack of molar teeth, children under the age of three are more susceptible to FB aspiration [3,4]. This unique anatomy can make the removal of FBs even more challenging [6].

The most common aspirated FBs are organic materials, such as peanuts, seeds, and nuts [1,2]. Inorganic objects typically aspirated are usually plastic or metal, including pieces of toys, coins, jewelry, magnets, or batteries [7,8]. The size of the FBs plays a role in how patients present. Larger objects can cause more severe symptoms if there is complete obstruction, whereas smaller objects can cause partial obstruction of the airway and present with minor signs and symptoms [3].

Chest X-rays can be helpful in diagnosing aspirated tracheobronchial FBs if the object is radiopaque; however, only about 3-34.3% of all FBs are radiopaque [9]. If a chest X-ray is not sufficient, a chest computed tomography (CT) scan can be considered for detecting radiopaque objects [9].

FBs should be removed immediately, as any retained objects can cause complications, including recurrent respiratory infections, persistent coughing or wheezing, pneumonia, bronchiectasis, lung consolidation, and abscess [2,4]. Within the pediatric population, definitive management of tracheobronchial FBs typically requires rigid bronchoscopy under general anesthesia, as it allows visualization of the trachea and proximal bronchi, and facilitates FB removal with direct visualization [3,4,6]. However, the removal of FBs may require additional instruments, such as baskets, forceps, and balloons [1]. While the choice of instrumentation varies based on equipment availability, subspecialist training also plays a role. This highlights the importance of a multidisciplinary approach for the removal of tracheobronchial FBs, which can help circumvent the need for invasive surgical removal [2].

This case report discusses a nine-year-old male who aspirated two ball magnets into the lower bronchial tree of the right lung and required a multidisciplinary approach for retrieval. Aspiration and removal of this specific object have not been reported in the pediatric population at this time.

## Case presentation

An otherwise healthy nine-year-old male presented to the Emergency Department for evaluation after swallowing two FBs, reported to be ball magnets, which he regurgitated from the esophagus and then subsequently aspirated into the lungs. Upon presentation, the patient was able to talk and breathe comfortably and confirmed the objects he had swallowed. He denied any associated shortness of breath, chest pain, abdominal pain, vomiting, or difficulty breathing.

**Figure 1:**
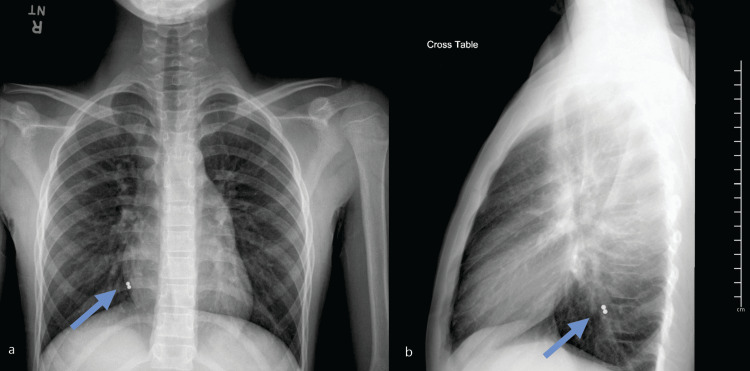
(a) X-ray AP chest: Two small circular radio-opacities (arrows), most likely ingested magnets, are seen in the right lower lobe. Hyperinflation of the lungs is noted, without consolidation. (b) X-ray lateral chest view of the circular radio-opacities. Image credit: Dr. Lena Naffaa

Initial workup included an X-ray of the neck, chest, and abdomen for evaluation of ingested FBs, which showed two tiny circular radio-opacities, assumed to be ingested magnets, in the right lower lobe of the lungs, with some slight bilateral hyperinflation and no focal lung consolidation, pleural effusion, or pneumothorax seen (Figures 1a-1b).

**Figure 2:**
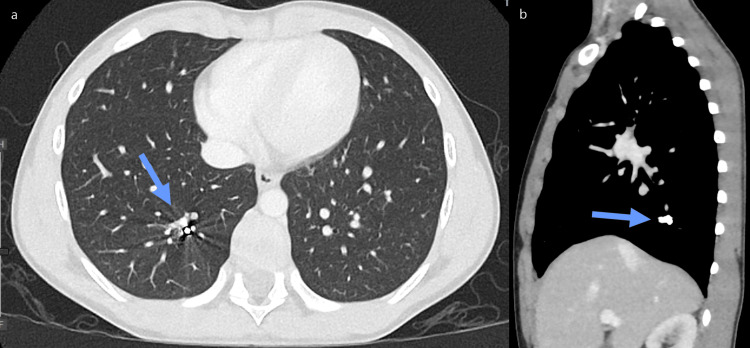
(a) Axial CT of the chest: Two small circular radio-opacities (arrows) located in the posterior basal segment of the right lower lobe. (b) Sagittal CT chest. Image credit: Dr. Lena Naffaa CT: Computed tomography

A CT scan of the chest with contrast was requested by otolaryngology (ENT) for intraoperative planning. The chest CT showed that the two FBs were located in the posterior basal segment of the right lower lobe. Although an exact location could not be determined, they were thought to be in the distal branches of the right lower lobe posterior segmental branching system (Figures 2a-2b).

After a review of all imaging, the patient was taken to the operating room (OR) with pulmonology, ENT, general surgery, and interventional radiology present. A multidisciplinary approach was required for removal due to the challenging location of the FBs.

**Figure 3:**
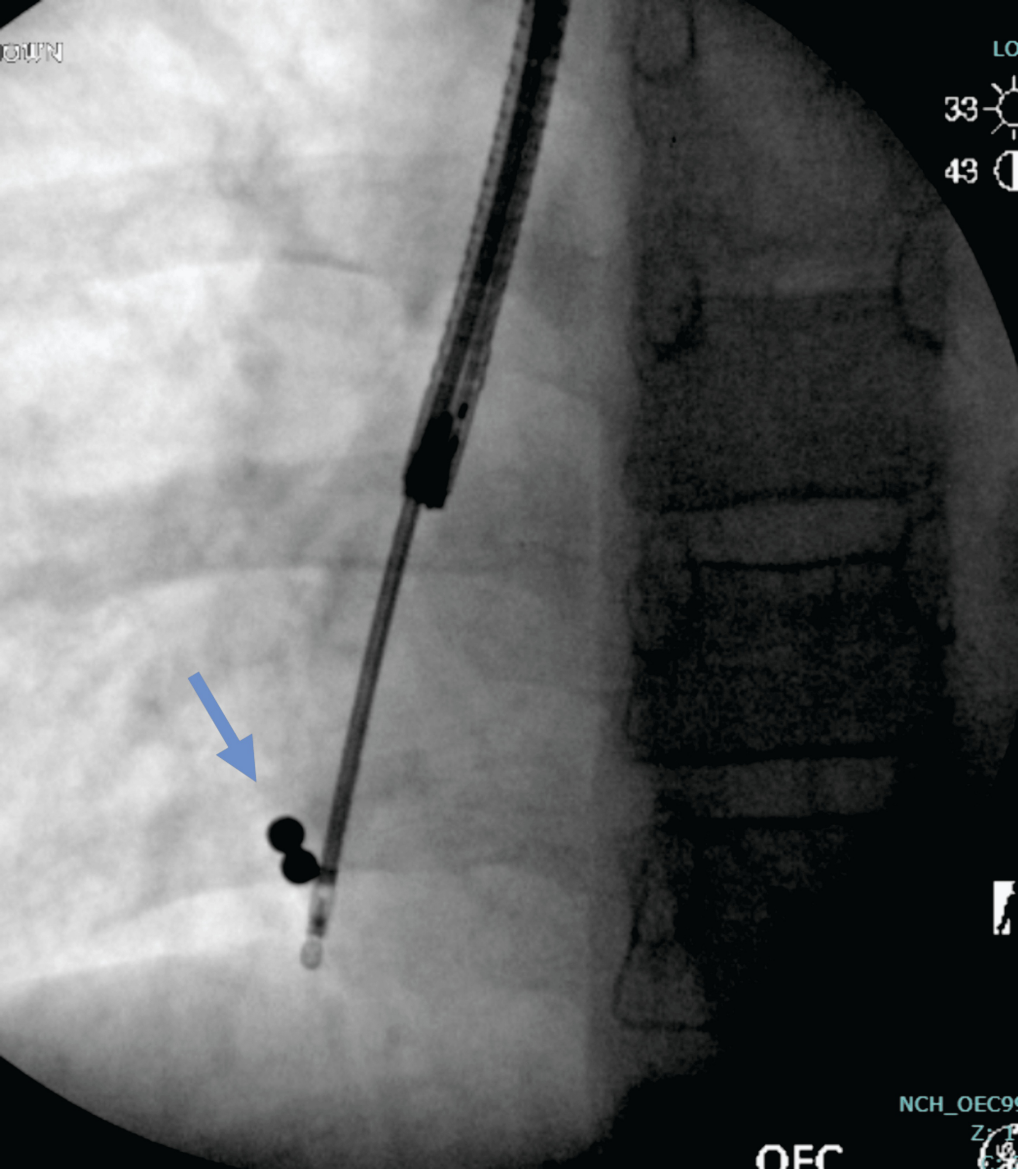
AP chest view of the right chest during the fluoroscopic removal of two small circular radio-opacities (arrow). Image credit: Dr. Lena Naffaa

**Figure 4:**
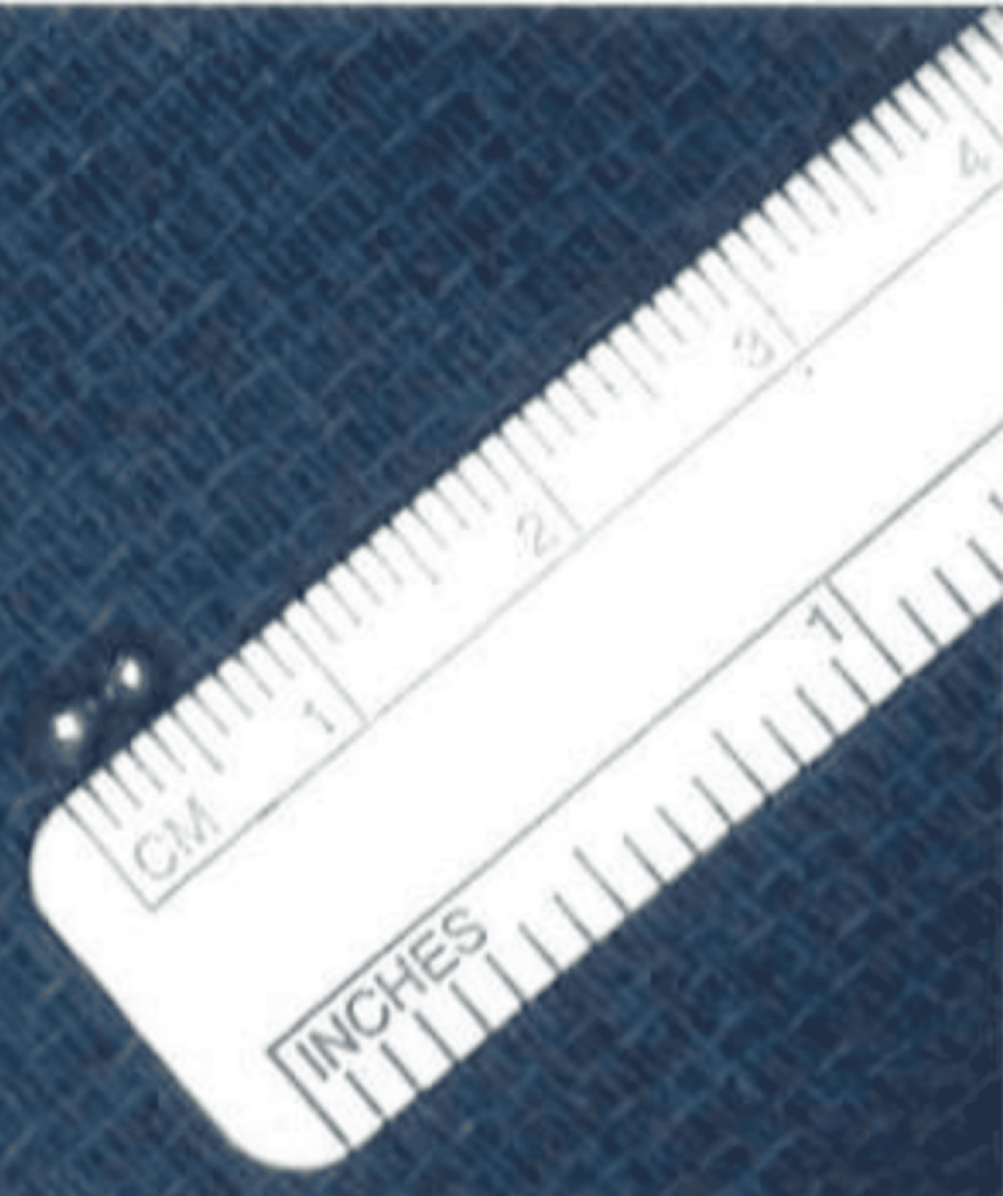
Retrieved foreign bodies: two round ball magnets, approximately 2.5 mm each.

Flexible bronchoscopy was performed via a 4.1 mm scope (2.0 channel) by pulmonology and ENT. Extensive exploration of the right middle lobe and right lower lobe, including the subsegments, was conducted with the assistance of fluoroscopy to locate the FBs (Figure 3). Two round FBs were visualized in the distal branch of the posterior basal segment of the right lower lobe, with some surrounding mucus. Upon visualization, both magnets were found together within the same segment. Both FBs were then retrieved using a metal raptor grasping device, which was threaded through the flexible bronchoscope channel, and were confirmed to be two round ball magnets, approximately 2.5 mm each (Figure 4). A final chest X-ray was performed to confirm that all FBs were removed and that the lungs were normally expanded and clear, without pneumothorax or pneumomediastinum.

Throughout the entire process, general surgery assisted with the utilization of fluoroscopy, and the team worked together on instrument selection for the removal of the magnets. Additionally, general surgery remained present in the OR in case the patient experienced a pulmonary hemorrhage or pneumothorax during the removal process, or if removal via bronchoscopy failed, requiring operative retrieval.

After the successful removal of the two ball magnets via flexible bronchoscopy, the patient was admitted overnight for observation and did not have any subsequent complications. He was discharged home on postoperative day 1 with a course of systemic steroids, and albuterol and budesonide treatments twice daily for five days.

## Discussion

Airway FBs present one of the most common life-threatening airway emergencies in children [3,4]. Within the pediatric population, tracheobronchial FBs are among the leading causes of mortality, and bilateral FBs within the airway can cause even higher mortality, possibly leading to suffocation or death [4,5,7]. Due to their narrower and shorter upper airway anatomy, an underdeveloped protective airway mechanism, and a lack of molar teeth, children under the age of three are more susceptible to FB aspiration [3,4]. Children in this age group are also inclined to explore their environment and place objects in their mouths while eating, crying, and playing [3]. Beyond this age, other factors such as trauma, abuse, neurological dysfunction, and psychological disorders can lead to tracheobronchial FB aspiration [1]. Additionally, as seen with our patient, adolescents can also play with objects in their mouths due to influence from their peers. This patient was attempting to separate these magnets with his teeth for his friends when an accidental aspiration occurred.

Most FBs enter the right main bronchus due to vertical anatomy and a larger diameter, but they can lodge anywhere from the supraglottis to the terminal bronchioles [1,3]. Approximately 80-90% of aspirated FBs get stuck in the bronchi due to their configuration and size [7]. In children, their narrow anatomy can make removal even more difficult [6].

Aspirated FBs are either organic or inorganic. One study found peanuts to be the most common aspirated organic FBs [3]. Inorganic objects typically aspirated are usually plastic or metal pieces, such as toys, coins, jewelry, magnets, or batteries [7,8]. The size of the FBs plays a role in how patients present, as larger objects can cause complete obstruction and more severe symptoms, whereas smaller objects usually cause partial obstruction of the airway and present with less severe signs and symptoms [3]. The severity of symptoms varies with regard to the type of FBs, where the FBs are lodged, the degree of obstruction, and how long the FBs have been within the tracheobronchial tree [4,7]. Typically, symptoms include wheezing, stridor, decreased breath sounds on auscultation, collapse or hyperinflation of the affected lung, and recurrent pneumonia for FBs that have been there longer [4]. One study indicated that the most common symptom is coughing [3]. Additionally, symptoms and the severity of symptoms can also be impacted by delayed diagnosis if FB aspiration is unwitnessed. Delayed diagnosis can result in atypical patient presentation, which includes complications such as recurrent pneumonia, emphysema, bronchiectasis, and potentially death [4]. Sometimes, there may not be any symptoms at all, especially if there is an incomplete blockage of the respiratory tract [1,2].

Chest X-rays can be helpful in diagnosing aspirated tracheobronchial FBs if the object is radiopaque; however, these are not always sensitive modalities, as only approximately 3-34.3% of all FBs are radiopaque [9]. A chest X-ray may also show indirect findings, such as consolidation, hyperinflation, atelectasis, and pneumothorax or pneumomediastinum, which are both rare findings [2]. If a chest X-ray is not sufficient, a chest CT scan can be considered, as it can detect radiopaque objects and also show signs of pulmonary complications in patients with delayed diagnosis of aspirated FBs [8,9].

FBs should be removed immediately, as any retained FBs can cause complications, including recurrent respiratory infections, persistent coughing or wheezing, pneumonia, bronchiectasis, lung consolidation, and abscess [2,4]. Retained tracheobronchial FBs can also cause tissue reaction, resulting in granulation tissue formation and bronchovascular or bronchopleural fistulas [1].

Within the pediatric population, definitive management of tracheobronchial FBs typically requires rigid bronchoscopy under general anesthesia, as it allows visualization of the trachea, proximal bronchi, and direct FB removal with visualization [3,4,6]. Rigid bronchoscopy is also the most sensitive and specific method for diagnosing and treating aspirated tracheobronchial FBs [1]. Studies have shown a high success rate for the removal of tracheobronchial FBs with flexible bronchoscopy (87.1%), but rigid bronchoscopy has a higher success rate (95-99%) [2]. However, the removal of FBs may require additional instruments, such as baskets, forceps, and balloons [1]. While the choice of instrumentation varies based on equipment availability, subspecialist training also plays a role. For example, pulmonologists are primarily trained in flexible bronchoscopy, while otolaryngologists are trained in both flexible and rigid bronchoscopy. Additionally, removing FBs that are more distal within the tracheobronchial tree is more challenging and may require the combined use of rigid and flexible bronchoscopes, as well as additional guidance with fluoroscopy [1].

A case involving a one-year-old male who had an FB lodged in the distal bronchus utilized recommendations from anesthesia to remove the FB. Initially, an attempt to remove the FB was made with a Fogarty catheter and basket forceps, but due to the different diameters of the rigid bronchoscope and the patient’s bronchus where the FB was located, the anesthesiologists recommended using a suction catheter with a thinner flexible bronchoscope for removal, which was successful [6]. This highlights the importance of a multidisciplinary approach for the removal of tracheobronchial FBs, and this type of management can help circumvent the need for invasive surgical procedures [2].

## Conclusions

FB aspiration within the pediatric population is one of the most common life-threatening airway emergencies. It is important to be aware of the different types of objects that can be aspirated and to understand that a multidisciplinary approach can contribute to successful removal without the need for invasive surgical intervention. It is also prudent to recognize that children can aspirate all types of objects, which may require a novel approach for removal. This case highlights a previously unreported object being aspirated into the lower lobe of the right lung, which required the efforts of multiple subspecialties for nonsurgical removal.
